# Downregulation of Ca^2+^-Activated Cl^−^ Channel TMEM16A Mediated by Angiotensin II in Cirrhotic Portal Hypertensive Mice

**DOI:** 10.3389/fphar.2022.831311

**Published:** 2022-03-16

**Authors:** Rubii Kondo, Nami Furukawa, Akari Deguchi, Naoki Kawata, Yoshiaki Suzuki, Yuji Imaizumi, Hisao Yamamura

**Affiliations:** Department of Molecular and Cellular Pharmacology, Graduate School of Pharmaceutical Sciences, Nagoya City University, Nagoya, Japan

**Keywords:** TMEM16A, calcium-activated chloride channel, portal hypertension, portal vein, cirrhosis, angiotensin II, bilirubin, smooth muscle

## Abstract

Portal hypertension is defined as an increased pressure in the portal venous system and occurs as a major complication in chronic liver diseases. The pathological mechanism underlying the pathogenesis and development of portal hypertension has been extensively investigated. Vascular tone of portal vein smooth muscles (PVSMs) is regulated by the activities of several ion channels, including Ca^2+^-activated Cl^−^ (Cl_Ca_) channels. TMEM16A is mainly responsible for Cl_Ca_ channel conductance in vascular smooth muscle cells, including portal vein smooth muscle cells (PVSMCs). In the present study, the functional roles of TMEM16A channels were examined using two experimental portal hypertensive models, bile duct ligation (BDL) mice with cirrhotic portal hypertension and partial portal vein ligation (PPVL) mice with non-cirrhotic portal hypertension. Expression analyses revealed that the expression of TMEM16A was downregulated in BDL-PVSMs, but not in PPVL-PVSMs. Whole-cell Cl_Ca_ currents were smaller in BDL-PVSMCs than in sham- and PPVL-PVSMCs. The amplitude of spontaneous contractions was smaller and the frequency was higher in BDL-PVSMs than in sham- and PPVL-PVSMs. Spontaneous contractions sensitive to a specific inhibitor of TMEM16A channels, T16A_inh_-A01, were reduced in BDL-PVSMs. Furthermore, in normal PVSMs, the downregulation of TMEM16A expression was mimicked by the exposure to angiotensin II, but not to bilirubin. This study suggests that the activity of Cl_Ca_ channels is attenuated by the downregulation of TMEM16A expression in PVSMCs associated with cirrhotic portal hypertension, which is partly mediated by increased angiotensin II in cirrhosis.

## Introduction

Portal hypertension is characterized by a pathological increase in pressure in the portal venous system (Bandali et al., 2017). It is a common and severe complication of chronic liver diseases. The most frequent cause is cirrhosis. Hepatic fibrosis due to cirrhosis causes an increase in hepatic vascular tone and morphological changes occurring during vascular remodeling. Increased hepatic resistance leads to portal hypertension and subsequent disturbances in the splanchnic and systemic circulations, resulting in serious symptoms such as gastroesophageal varices, variceal hemorrhage, splenomegaly, ascites, and hepatic encephalopathy (Bosch et al., 2009; García-Pagán et al., 2012). The normal portal venous pressure is 5–10 mmHg (Bandali et al., 2017) and the hepatic venous pressure gradient (HVPG), which is a normal pressure gradient between the portal vein and the inferior vena cava, is typically 1–5 mmHg (Bosch et al., 2009). Currently, HVPG measurement is the gold-standard method to evaluate the presence and severity of portal hypertension. Clinically significant portal hypertension is defined as an increase in HVPG to ≥10 mmHg (Bosch et al., 2009). The molecular mechanism underlying the pathogenesis and development of portal hypertension has been discussed from many functional aspects.

Vascular tone and pressure are mainly regulated by the cytosolic Ca^2+^ concentration ([Ca^2+^]_cyt_) under the physiological and pathological conditions. Following vasoconstrictor stimulation and membrane depolarization, an increase in [Ca^2+^]_cyt_ is caused by two major pathways: 1) Ca^2+^ influx through the voltage-dependent Ca^2+^ channels (VDCCs) and receptor-operated Ca^2+^ (ROC) channels in the plasma membrane, and 2) Ca^2+^ release from the intracellular Ca^2+^ store sites such as the sarcoplasmic reticulum (SR) and mitochondria (Chalmers et al., 2007). Increased [Ca^2+^]_cyt_ in vascular smooth muscles facilitates excitation-contraction coupling. In parallel, it stimulates Ca^2+^-activated ion channels in the plasma membrane and regulates vascular tone. For example, the activation of Ca^2+^-activated K^+^ (K_Ca_) channels causes a membrane hyperpolarization and reduces the activity of VDCCs (Latorre et al., 2017). On the other hand, the opening of Ca^2+^-activated Cl^−^ (Cl_Ca_) channels causes membrane depolarization and increases the VDCC activity (Kitamura and Yamazaki, 2001; Bulley and Jaggar, 2014). Therefore, the balance between K_Ca_ and Cl_Ca_ channel conductance determines the membrane excitability, myogenic tone, contraction/relaxation, and pressure in vascular smooth muscles.

Cl_Ca_ channels are involved in many physiological processes such as smooth muscle contraction, epithelial secretion, cell volume regulation, neuronal signaling, and sensory transduction (Pedemonte and Galietta, 2014). In vascular smooth muscles, Cl_Ca_ channels play significant roles in the regulation of membrane excitability, myogenic tone, and muscle contraction (Pedemonte and Galietta, 2014). So far, TMEM16A has been identified as a molecular entity responsible for Cl_Ca_ channels in vascular smooth muscles (Pedemonte and Galietta, 2014), including portal veins (Davis et al., 2010; Ohshiro et al., 2014a; Ohshiro et al., 2014b). Under physiological conditions, the activation of TMEM16A Cl_Ca_ channels shifts the resting membrane potential to the positive direction and promotes Ca^2+^ influx through VDCCs, resulting in an increase in vascular tone and pressure (Kitamura and Yamazaki, 2001; Bulley and Jaggar, 2014). On the other hand, there is limited information on the pathological role of TMEM16A Cl_Ca_ channels in cardiovascular diseases. A recent report showed that TMEM16A channels regulate the proliferation of portal vein smooth muscle cells (PVSMCs) in portal hypertension (Zeng et al., 2018).

The present study was performed to elucidate the involvement of TMEM16A Cl_Ca_ channels in the pathological mechanism of portal hypertension, using expression analyses, electrophysiological recordings, and contractility measurements. Two different portal hypertensive animal models, bile duct ligation (BDL) mice as a portal hypertensive model resulting from cirrhosis and partial portal vein ligation (PPVL) mice as an idiopathic portal hypertensive model without hepatic dysfunction (Abraldes et al., 2006; Geerts et al., 2008; Bosch and Iwakiri, 2018), were prepared to analyze the functional expression of TMEM16A channels in PVSMCs. The expression and function of TMEM16A channels were downregulated in BDL-PVSMCs compared with sham- and PPVL-PVSMCs, which was partly mediated by angiotensin II, the serum concentration of which increases in patients with cirrhosis.

## Materials and Methods

### Ethical Approval

All experiments were approved by the Ethics Committee of Nagoya City University (H30-P-1) and were conducted in accordance with the Guide for the Care and Use of Laboratory Animals of the Japanese Pharmacological Society.

### BDL Operation

Male mice (C57BL/6, 8-week-old; Japan SLC, Hamamatsu, Japan) were anesthetized under isoflurane (Wako Pure Chemical Industries, Osaka, Japan) inhalation. After the midline abdominal incision, the common bile duct was occluded with a double ligature using a suture (nylon 8-0; Alfresa Pharma, Osaka, Japan). The first ligature was made below the junction of the hepatic ducts and the second ligature was made above the entrance of the pancreatic ducts. The portion of the bile duct between the two ligatures was cut to avoid re-permeabilization. In sham-operated mice, the abdominal cavity was opened and the common bile duct was isolated, but there was no ligation. Experiments were performed at 4–5 weeks after sham or BDL operation (Abraldes et al., 2006; Geerts et al., 2008; Bosch and Iwakiri, 2018).

### PPVL Operation

Under isoflurane inhalation anesthesia, a midline abdominal incision was made and the portal vein was separated from the surrounding tissues. A ligature (nylon 6-0) was tied around both portal vein and a blunt-tipped 27-gauge needle. Subsequent removal of the needle yielded calibrated stenosis of the portal vein. In sham-operated mice, the abdominal cavity was opened and the portal vein was separated, but there was no ligation. Experiments were performed at 2–3 weeks after sham or PPVL operation (Abraldes et al., 2006; Geerts et al., 2008; Bosch and Iwakiri, 2018).

### Real-Time PCR

Total RNA extraction from homogenates of murine portal vein smooth muscles (PVSMs), reverse transcription, and real-time PCR using the ABI PRISM 7000 (Applied Biosystems, Foster City, CA, United States) and LightCycler 96 (Roche Diagnostics, Mannheim, Germany) real-time PCR systems, were performed as reported previously (Ohshiro et al., 2014a; Yamamura et al., 2018). Specific primers for murine TMEM16 genes were designed as follows: TMEM16A (GenBank Accession number, NM_178642), (+) GAT CTC CTT CAC GTC TGA CTT CAT C, (−) TGC TGT GCC ATT CTG GAA G; TMEM16B (NM_153589), (+) CTT TAT CCC CCG CCT TGT GTA, (−) TTC AGG CTG TGT TCC CTC CTT; TMEM16C (NM_001128103), (+) AAT TGC CTA AAG GGC TAT GTC AAC, (−) TCC AAG GTG GGC CTC TAT AGT CT; TMEM16D (NM_178773), (+) ACT GCA GTT CTG GCA TGT TCT C, (−) CTT TCG GGA GGT CTG GTA TCA G; TMEM16E (NM_177694), (+) GCC CTT GAG TGG ATA CGT CAA TA, (−) TGC AGG TGA CGA AGT CTT TTT TC; TMEM16F (NM_175344), (+) CCC ATA CAT TGG GCT TGG TAA, (−) CAC GTG CCA ATA GTA GAT GTT GTG; TMEM16G (NM_207031), (+) TTC CTG CCA CGT GTC TAC TAC AG, (−) GTT GTG TGC GGA GGT GAA AGT; TMEM16H (NM_001164679), (+) CAG GAC TAC CAG GAG ATG TTC GT, (−) TCG GAT CTC AAT CAG GTT GTT G; TMEM16J (NM_178381), (+) GGT ACC GGG ACT ACC GAA ATG, (−) GGC AAA ATG CTC AAA GAG GAT AAC; TMEM16K (NM_133979), (+) CCT TGA AAA TGT GCA GGG TCT T, (−) CCA CGG ATA TAA CGC TCA TCG T; and GAPDH (NM_008084), (+) CAT GGC CTT CCG TGT TCC T, (−) CCT GCT TCA CCA CCT TCT TGA. Values for each unknown sample relative to the standard curve for specific primers were calculated and yielded transcriptional quantitation of gene products relative to the endogenous standard (GAPDH).

### Cell Isolation

The portal veins were dissected from mice and the endothelium was stripped out by water flow, as reported previously (Saleh and Greenwood, 2005; Ohshiro et al., 2014a). The PVSMs were incubated in physiological saline solution {PSS; 125 mM NaCl, 5.4 mM KCl, 0.05 mM CaCl_2_, 15.4 mM NaHCO_3_, 0.33 mM Na_2_HPO_4_, 0.34 mM KH_2_PO_4_, 10 mM glucose, and 11 mM 2-[4-(2-hydroxyethyl)-1-piperazinyl]ethanesulfonic acid (HEPES)} containing 0.3% protease (type XIV; Sigma-Aldrich, St. Louis, MO, United States) for 5 min at 37°C. The tissues were then incubated in PSS containing 0.6% collagenase (type IA; Sigma-Aldrich) for 5 min at 37°C. After incubation, these were dispersed mechanically in PSS.

### Immunocytochemistry

Immunocytochemical staining was performed as reported previously (Yamamura et al., 2012; Yamamura et al., 2018). In brief, freshly-isolated murine PVSMCs were fixed with 4% paraformaldehyde in phosphate-buffered saline (PBS) (-) (137 mM NaCl, 2.68 mM KCl, 8.1 mM Na_2_HPO_4_, and 1.47 mM KH_2_PO_4_) for 30 min at room temperature (23–25°C) in a multi-well glass bottom dish (Matsunami Glass, Osaka, Japan). These cells were treated with a polyclonal anti-TMEM16A antibody (1:500; ab191040, Abcam, Cambridge, MA, United States) and anti-α-smooth muscle actin (α-SMA, 1:500; #48938, Cell Signaling Technology, Danvers, MA, United States) for 12 h at 4°C, and then covered with Alexa Fluor 488- and Alexa Fluor 633-labeled secondary antibody solutions (1:1,000; A11008 and A21050, respectively, Molecular Probes-Invitrogen, Eugene, OR, United States) for 1 h at room temperature. Confocal images (single x-y images [resolution = 0.21 μm/pixel] from one focal plane [z = 2.0 μm]) of all samples were taken at the same time with same parameters by a confocal laser scanning microscopy system (A1R; Nikon, Tokyo, Japan) equipped with an inverted microscope (Eclipse Ti; Nikon), an objective lens (Plan Apo VC 60×/NA 1.40, oil immersion; Nikon), a solid-state 488-nm laser (laser power 3.0%, PMT HV 85; Sapphire 488 nm/20 mW, Coherent, Wilsonville, OR, United States), a diode 640-nm laser (laser power 2.0%, PMT HV 80; Cube 640 nm/40 mW, Coherent), and NIS Elements software (version 3.22; Nikon).

### Electrophysiological Recording

Electrophysiological recordings were performed on single murine PVSMCs using a whole-cell patch clamp technique with a CEZ-2400 amplifier (Nihon Kohden, Tokyo, Japan), analog-digital converter (Digidata 1440A; Axon-Molecular Devices, Foster City, CA, United States), and pCLAMP software (version 10; Axon-Molecular Devices) as previously reported (Yamamura et al., 2012; Yamamura et al., 2018). HEPES-buffered solution was used as an extracellular solution: 137 mM NaCl, 5.9 mM KCl, 2.2 mM CaCl_2_, 1.2 mM MgCl_2_, 14 mM glucose, and 10 mM HEPES. The pH was adjusted to 7.4 with NaOH. The pipette solution had the following ionic composition: 120 mM CsCl, 20 mM tetraethylammonium (TEA)-Cl, 2.8 mM MgCl_2_, 2 mM ATPNa_2_, 10 mM HEPES, 5 mM *O,O′*-bis(2-aminoethyl)ethyleneglycol-*N,N,N′,N′*-tetraacetic acid (EGTA), and 4.25 mM CaCl_2_ (pCa 6.0). The pH was adjusted to 7.2 with CsOH. Electrophysiological recordings were performed at room temperature.

### Contractility Measurements

Dissected portal veins were placed in aerated Krebs’ solution. A small piece of portal vein (5 mm long) was prepared and placed in an organ bath containing aerated Krebs’ solution at 37°C. One end of the segment was pinned to a rubber plate at the bottom of the bath and the other end was connected to an isometric transducer. The strips were stretched to 1 mN of tension. The Krebs’ solution had an ionic composition of 112 mM NaCl, 4.7 mM KCl, 2.2 mM CaCl_2_, 1.2 mM MgCl_2_, 25 mM NaHCO_3_, 1.2 mM KH_2_PO_4_, and 14 mM glucose. The pH was adjusted to 7.4 by gassing with a mixture of 95% O_2_ and 5% CO_2_.

### Organ Culture

Dissected portal veins were incubated in Dulbecco’s modified Eagle’s medium (DMEM; Wako Pure Chemical Industries) supplemented with 10% fetal bovine serum (FBS; Nichirei Biosciences, Tokyo, Japan), 100 U/ml of penicillin (Wako Pure Chemical Industries), and 10 μg/ml of streptomycin (Wako Pure Chemical Industries) in the absence (vehicle) and presence of the drug for 24 h at 37°C.

### Drugs

Pharmacological reagents were obtained from Sigma-Aldrich, except for bilirubin, TEA (Tokyo Chemical Industry, Tokyo Japan), EGTA, and HEPES (Dojindo Molecular Technologies, Kumamoto, Japan). T16A_inh_-A01 (2-[(5-ethyl-1,6-dihydro-4-methyl-6-oxo-2-pyrimidinyl)thio]-N-[4-(4-methoxyphenyl)-2-thiazolyl]-acetamide) and bilirubin were dissolved in dimethyl sulfoxide (DMSO) and 0.1 M NaOH, respectively, at a concentration of 30 mM as a stock solution.

### Statistical Analysis

Pooled data are shown as the mean ± S.E. The significance of differences between two groups was assessed by the Student’s *t*-test using BellCurve for Excel software (version 3.10; Social Survey Research Information, Tokyo, Japan). A *p* value of <0.05 was considered to be significant.

## Results

### Microscopic Features of Portal Hypertensive Mice

**FIGURE 1:**
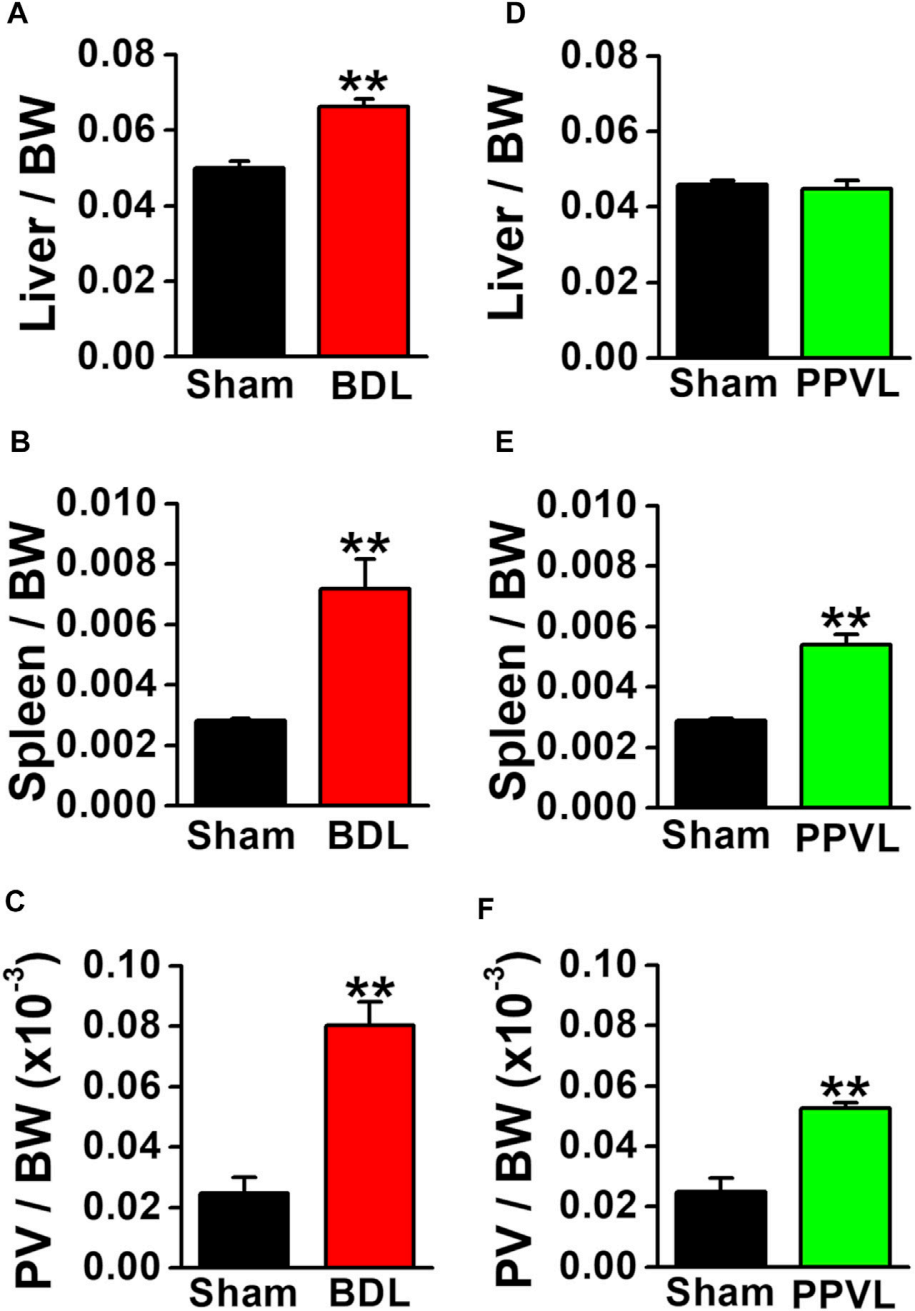
Macroscopic features of portal hypertensive mice. Two portal hypertensive mice were used: BDL mice as a portal hypertensive model associated with cirrhosis and PPVL mice as an idiopathic portal hypertensive model independently of cirrhosis. Wet tissue weights of portal hypertensive mice were measured and normalized by body weight (BW). **(A)** Liver weight in sham-operated and BDL mice (*n* = 12). **(B)** Spleen weight in sham-operated and BDL mice (*n* = 12–13). **(C)** Portal vein (PV) weight (5 mm long) in sham-operated and BDL mice (*n* = 5). **(D)** Liver weight in sham-operated and PPVL mice (*n* = 8–12). **(E)** Spleen weight in sham-operated and PPVL mice (*n* = 12–13). **(F)** Portal vein weight in sham-operated and PPVL mice (*n* = 3). Data are presented as means ± S.E. **p < 0.01 versus sham.

BDL mice are widely used as a portal hypertensive model associated with cirrhosis, whereas PPVL mice are used as an idiopathic non-cirrhosis portal hypertensive model (Abraldes et al., 2006; Geerts et al., 2008; Bosch and Iwakiri, 2018). BDL mice had yellow urine and skin. The liver weight increased at 4–5 weeks after BDL operation (0.066 ± 0.002 per body weight, *n* = 12, *p* < 0.001 versus sham, 0.050 ± 0.002, *n* = 12; Figure 1A). The spleen weight increased (0.0072 ± 0.0010 per body weight, *n* = 12, *p* < 0.001 versus sham, 0.0028 ± 0.0001, *n* = 13; Figure 1B). The portal vein weight (5 mm long) was also increased (0.080 ± 0.008 × 10^−3^ per body weight, *n* = 5, *p* < 0.001 versus sham, 0.025 ± 0.005 × 10^−3^, *n* = 5; Figure 1C). BDL mice exhibited jaundice, liver enlargement, splenomegaly, and portal vein hypertrophy. These macroscopic features represented the clinical symptoms of portal hypertension resulting from cirrhosis.

In contrast to BDL mice, the urine and skin colors were normal in PPVL mice. The liver weight at 2–3 weeks after PPVL operation (0.045 ± 0.002 per body weight, *n* = 8) was comparable with that in sham-operated mice (0.046 ± 0.001, *n* = 12; Figure 1D). On the other hand, the spleen weight increased (0.0050 ± 0.0003 per body weight, *n* = 13, *p* < 0.001 versus sham, 0.0029 ± 0.0001, *n* = 12; Figure 1E). The portal vein weight also increased (0.052 ± 0.002 × 10^−3^ per body weight, *n* = 3, *p* = 0.005 versus sham of 0.025 ± 0.004×10^−3^, *n* = 3). PPVL mice exhibited splenomegaly and portal vein hypertrophy, but not jaundice or liver enlargement. These macroscopic features represented the clinical symptoms of portal hypertension without hepatic dysfunction.

### Expression of TMEM16 Family in PVSMs From Portal Hypertensive Mice

**FIGURE 2:**
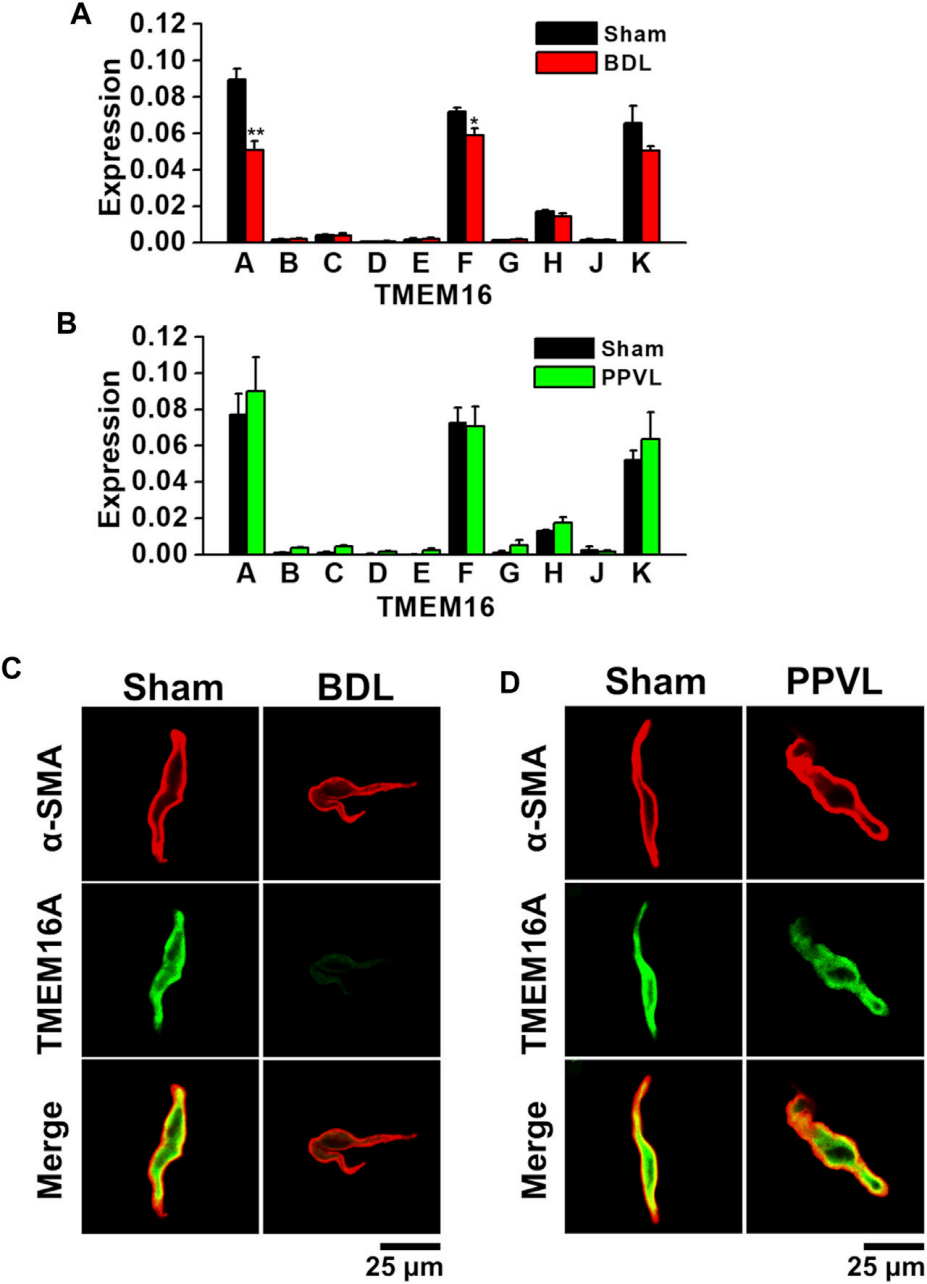
Downregulation of TMEM16A expression in PVSMCs from BDL mice. Expression level of TMEM16 channels in murine PVSMCs was analyzed by real-time PCR and immunocytochemistry. **(A)** Expression level of TMEM16 mRNAs in sham- and BDL-PVSMs (*n* = 4–6). The expression values were normalized to GAPDH. **(B)** Expression level of TMEM16 mRNAs in sham- and PPVL-PVSMs (*n* = 4–6). **(C)** Representative immunocytochemical staining of TMEM16A and α-SMA proteins in sham- and BDL-PVSMCs (*n* = 38–39). **(D)** Representative immunocytochemical staining of TMEM16A channels and α-SMA in sham- and PPVL-PVSMCs (*n* = 19–32). Data are presented as means ± S.E. *p < 0.05, **p < 0.01 versus sham.

The expression profiles of the TMEM16 family in PVSMs from portal hypertensive mice were analyzed using real-time PCR and immunocytochemical staining. Among the TMEM16 family, TMEM16A mRNA was expressed (0.090 ± 0.006 of GAPDH, *n* = 4) and TMEM16F and TMEM16K mRNAs were also detected (0.072 ± 0.002 and 0.066 ± 0.010, respectively, *n* = 4; Figure 2A) in sham-PVSMs, as reported previously (Ohshiro et al., 2014a). In BDL-PVSMs, the mRNA expression of TMEM16A was clearly reduced (0.051 ± 0.005, *n* = 6, *p* = 0.001). TMEM16F mRNA was slightly attenuated in BDL-PVSMs (0.059 ± 0.004, *n* = 6, *p* = 0.042), whereas TMEM16K mRNA was identical to that in sham-PVSMs (0.051 ± 0.002, *n* = 6). On the other hand, there were no significant differences in the expression of TMEM16 genes between sham- and PPVL-PVSMs (*n* = 4 to 6; Figure 2B).

Because the reduced expression of TMEM16A proteins in BDL-PVSMs was shown by Western blotting (Zeng et al., 2018), immunocytochemical staining was performed for protein expression analysis in the present study. Immunocytochemical staining can detect the localization of the protein in cells. Based on immunocytochemical staining, TMEM16A-coding proteins were abundantly expressed at the plasma membrane of sham-PVSMCs (*n* = 38), but it was clearly attenuated in BDL-PVSMCs (*n* = 39; Figure 2C). On the other hand, the distribution of TMEM16A proteins in PPVL-PVSMCs (*n* = 19) was almost the same as that in sham-PVSMCs (*n* = 32; Figure 2D). Taken together, TMEM16A expression was downregulated in BDL-PVSMCs at the mRNA and protein levels compared with that in sham- and PPVL-PVSMCs.

### Cl_Ca_ Currents in PVSMCs From Portal Hypertensive Mice

**FIGURE 3:**
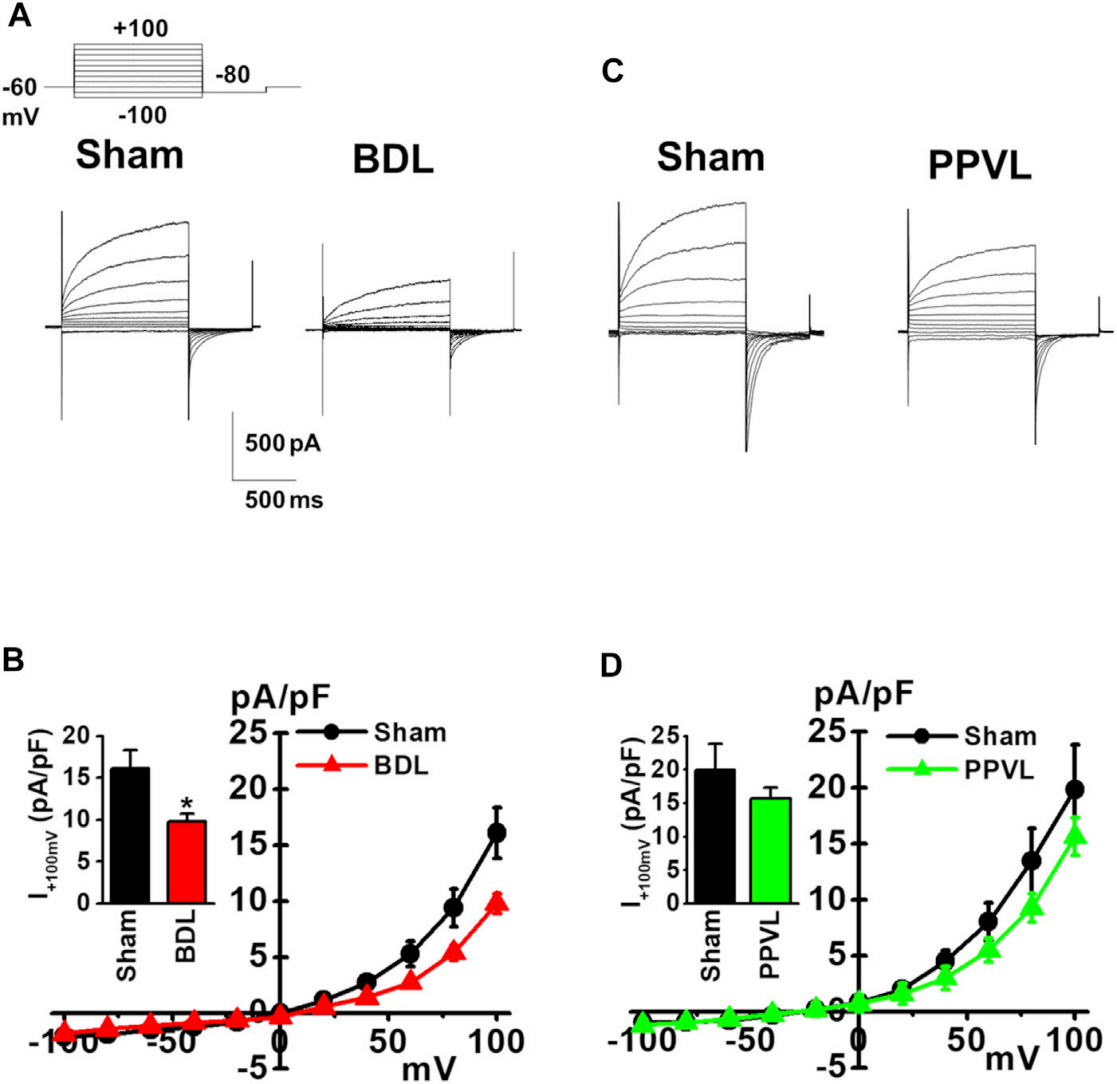
Reduced Cl_Ca_ currents in PVSMCs from BDL mice. Under whole-cell voltage-clamp configurations, freshly isolated PVSMCs were stimulated from a holding potential of −60 mV to selected test potentials (from −100 to +100 mV) by 20-mV increments for 1 s and then repolarized to −80 mV for 500 ms. **(A)** Representative traces of Cl_Ca_ currents in sham- and BDL-PVSMCs. **(B)** Current-voltage (I-V) relationship of Cl_Ca_ currents in sham- and BDL-PVSMCs. Peak amplitude of outward currents at +100 mV (inset; *n* = 7–12). **(C)** Representative traces of Cl_Ca_ currents in sham- and PPVL-PVSMCs. **(D)** Current-voltage (I-V) relationship of Cl_Ca_ currents in sham- and PPVL-PVSMCs. Peak amplitude of outward currents at +100 mV (inset; *n* = 5–7). Data are presented as means ± S.E. *p < 0.05 versus sham.

Cl_Ca_ currents were measured in freshly isolated murine PVSMCs using K^+^ deficient and Cl^−^ rich solutions containing TEA-Cl under whole-cell voltage-clamp conditions (see Materials and Methods). In addition, to activate Cl_Ca_ currents, the Ca^2+^ concentration in the pipette solution was fixed to pCa 6.0 (1 μM). Single PVSMCs were stimulated from the holding potential of −60 mV to selected test potentials (from −100 to +100 mV by 20-mV increments, every 15 s) for 1 s, and then repolarized to −80 mV for 500 ms. The cell capacitance of sham-PVSMCs was 34.9 ± 2.3 pF (*n* = 12). The depolarizing pulse elicited time-dependent outward currents at membrane potentials positive to +40 mV in sham-PVSMCs (16.1 ± 2.3 pA/pF at +100 mV, *n* = 12; Figures 3A,B). Upon repolarization, characteristic inward tail currents were recorded (−12.0 ± 1.8 pA/pF at −80 mV following +100 mV depolarization, *n* = 12). The current-voltage relationship demonstrated that the reversal potential was approximately 0 mV (*n* = 12). Our previous report showed that these currents were inhibited by 100 μM niflumic acid (a conventional Cl_Ca_ channel blocker) and 10–30 μM T16A_inh_-A01 (a specific TMEM16A channel blocker) (Ohshiro et al., 2014a).

Next, Cl_Ca_ currents were measured in BDL-PVSMCs. The cell capacitance was similar to that in sham-PVSMCs (40.5 ± 4.6 pF, *n* = 7). The outward currents were smaller in BDL-PVSMCs than in sham-PVSMCs (9.8 ± 0.9 pA/pF, *n* = 7, *p* = 0.022; Figures 3A,B). The inward currents were also reduced in BDL-PVSMCs (−6.9 ± 0.8 pA/pF, *n* = 7, *p* = 0.023). Similar to sham-PVSMCs, the current-voltage relationship demonstrated that the reversal potential was approximately 0 mV (*n* = 7). On the other hand, the amplitude of outward and inward Cl_Ca_ currents in PPVL-PVSMCs was similar to that in sham-PVSMCs (*n* = 5 to 7; Figures 3C,D). This suggested that, in BDL-PVSMCs, the activity of Cl_Ca_ currents was reduced by the downregulation of TMEM16A expression.

### Spontaneous Contractions in PVSMs From Portal Hypertensive Mice

**FIGURE 4:**
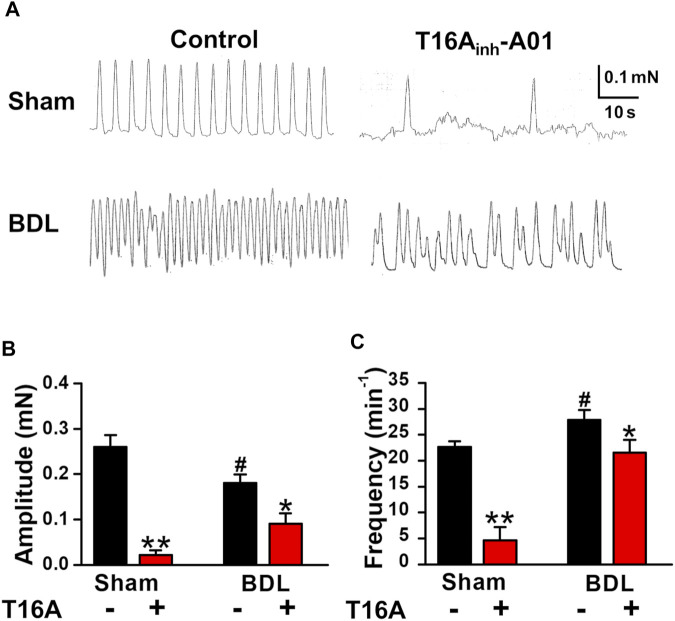
Attenuated spontaneous contraction in PVSMs from BDL mice. The characteristics of spontaneous contraction and effects of T16A_inh_-A01, a specific inhibitor of TMEM16A channels, on spontaneous contraction in PVSMs were analyzed in BDL mice. **(A)** Representative recordings of spontaneous contraction in the absence and presence of 30 μM T16A_inh_-A01 in sham- and BDL-PVSMs. **(B)** The amplitude of spontaneous contractions in the absence and presence of T16A_inh_-A01 (T16A) in sham- and BDL-PVSMs (*n* = 5–8). **(C)** The frequency of spontaneous contractions in the absence and presence of T16A_inh_-A01 (T16A) in sham- and BDL-PVSMs (*n* = 5–8). Data are presented as means ± S.E. *p < 0.05, **p < 0.01 versus control; ^#^ *p* < 0.05 versus sham/control.

It has been reported that portal veins exhibit spontaneous contractions sensitive to Cl_Ca_ blockers (Kirkup et al., 1996; Saleh and Greenwood, 2005). To clarify the involvement of TMEM16A channels in spontaneous contractions, the contractile responses were measured in PVSMs from portal hypertensive mice. In isometric tension experiments, sham-PVSMs were spontaneously active (Figure 4A). The amplitude of spontaneous contractions was 0.260 ± 0.026 mN (*n* = 8; Figure 4B) and the frequency was 22.7 ± 1.9 min^−1^ (*n* = 8; Figure 4C). The spontaneous contractions were mostly inhibited by a specific blocker of TMEM16A channels, 30 μM T16A_inh_-A01 (0.022 ± 0.010 mN, *n* = 5, *p* = 0.002 versus control and 4.6 ± 2.6 min^−1^, *n* = 5, *p* < 0.001, respectively).

**FIGURE 5:**
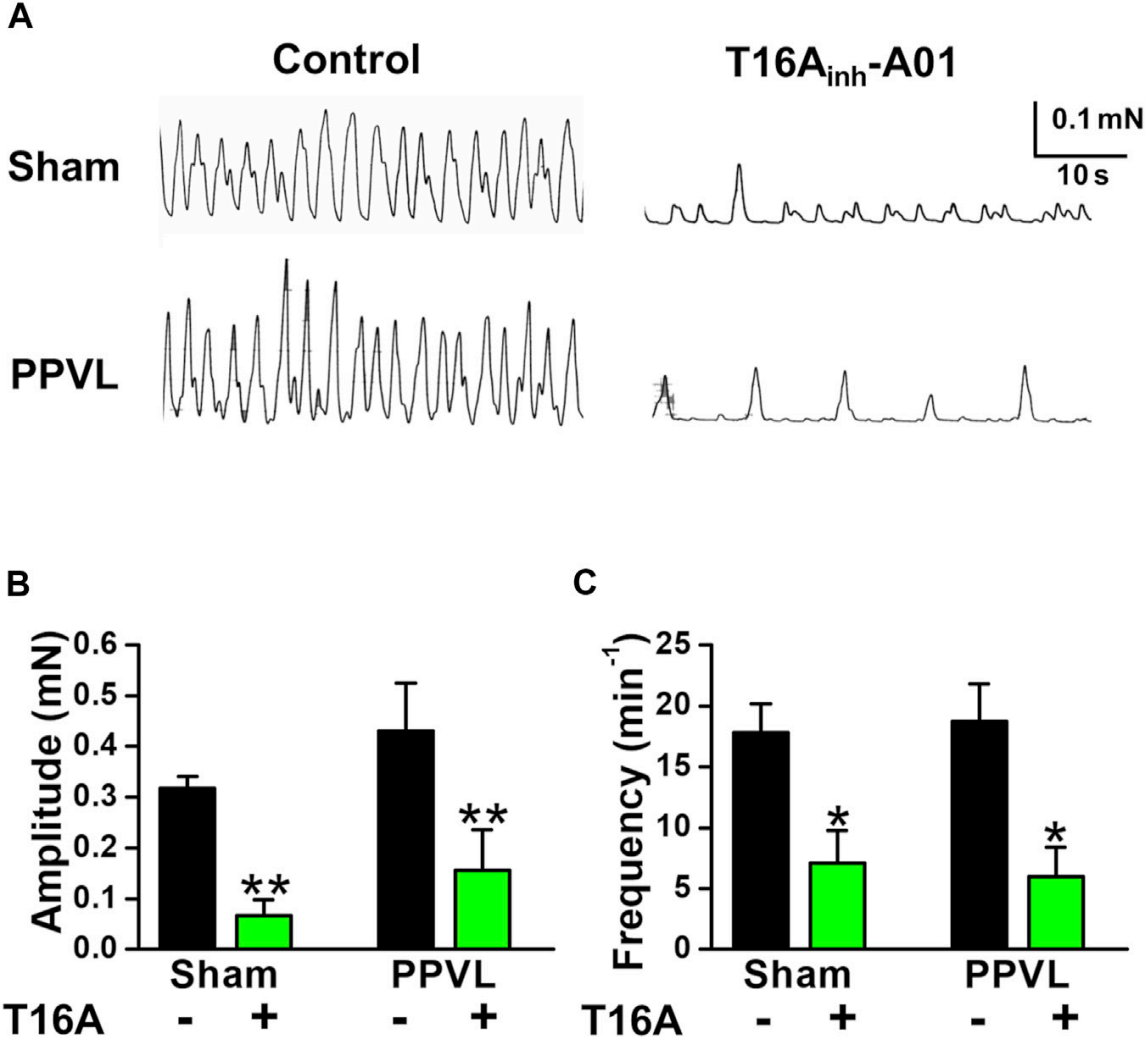
Spontaneous contraction in PVSMs from PPVL mice. The parameters of spontaneous contraction and effects of T16A_inh_-A01 on spontaneous contraction in PVSMs were examined in PPVL mice. **(A)** Representative recordings of spontaneous contraction in the absence and presence of 30 μM T16A_inh_-A01 in sham- and PPVL-PVSMs. **(B)** The amplitude of spontaneous contractions in the absence and presence of T16A_inh_-A01 (T16A) in sham- and PPVL-PVSMs (*n* = 5). **(C)** The frequency of spontaneous contractions in the absence and presence of T16A_inh_-A01 (T16A) in sham- and PPVL-PVSMs (*n* = 5). Data are presented as means ± S.E. *p < 0.05, **p < 0.01 versus control.

In BDL-PVSMs, the amplitude of spontaneous contractions was reduced (0.181 ± 0.018 mN, *n* = 8, *p* = 0.025; Figures 4A,B) and the frequency was slightly increased (27.9 ± 1.9 min^−1^, *n* = 8, *p* = 0.034; Figures 4A,C). Of note, the sensitivity to T16A_inh_-A01 was lower than that in sham-PVSMs (to 0.091 ± 0.023 mN, *n* = 5, *p* = 0.031 versus control, *p* = 0.007 versus sham; and 21.6 ± 2.4 min^−1^, *n* = 5, *p* = 0.026 versus control, *p* = 0.001 versus sham). The T16A_inh_-A01-insensitive component was inhibited by the further addition of a conventional Cl_Ca_ channel blocker, 100 μM niflumic acid (*n* = 4). On the other hand, spontaneous contractions were observed in PPVL-PVSMs to a similar degree as in sham-PVSMs (Figure 5A). The amplitude, frequency, and sensitivity to T16A_inh_-A01 in PPVL-PVSMs were similar to those in sham-PVSMs (*n* = 5; Figures 5B,C). This suggested that the downregulation of TMEM16A expression reduced spontaneous contractions sensitive to T16A_inh_-A01 in BDL-PVSMs.

### Effects of Bilirubin on the Expression and Activity of TMEM16A Channels in Murine PVSMCs

**FIGURE 6:**
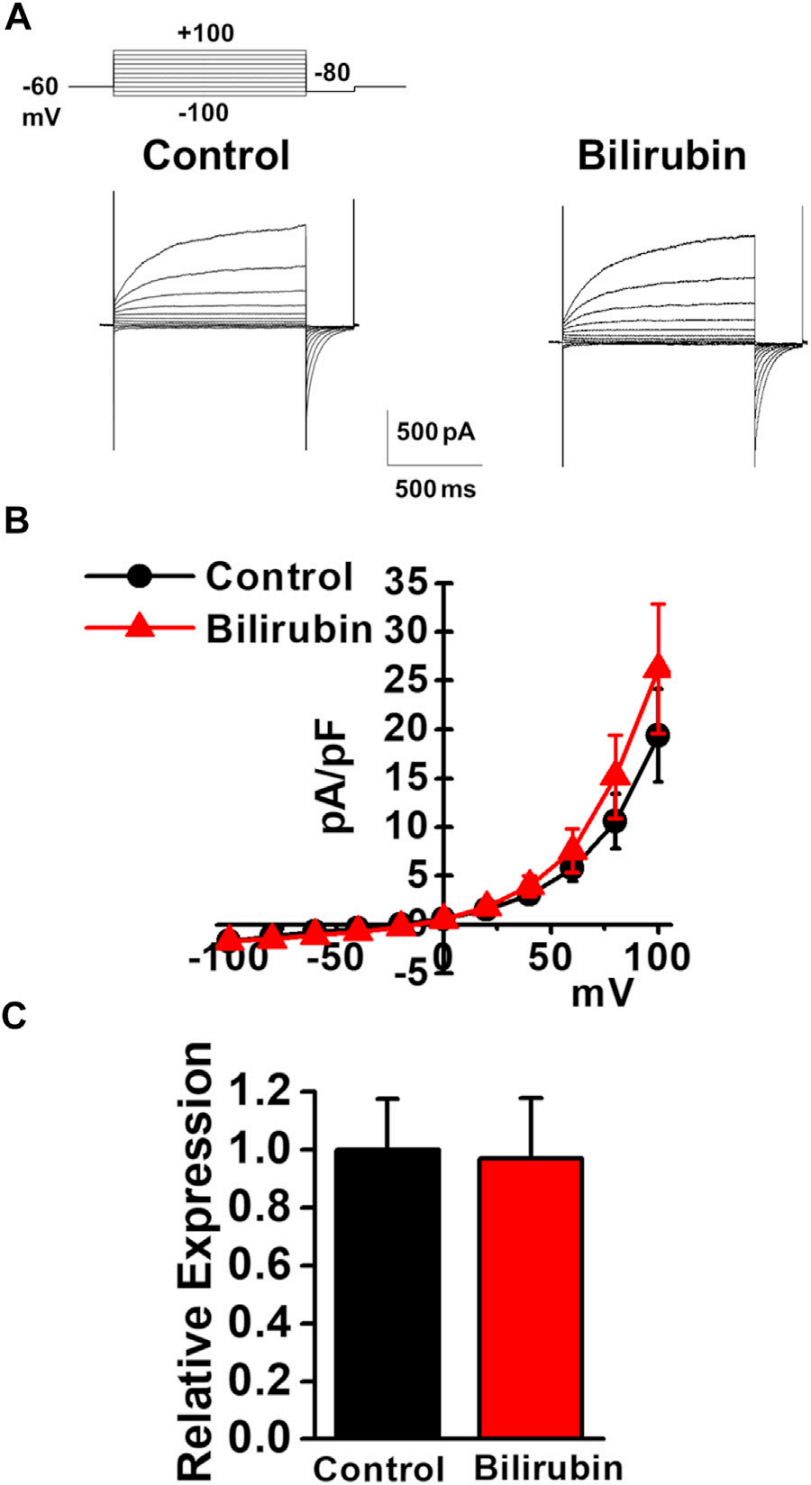
Effects of bilirubin on TMEM16A activity in murine PVSMCs. Effects of bilirubin on the functional expression of TMEM16A channels were examined in murine PVSMCs. **(A)** Representative traces of Cl_Ca_ currents in the absence and presence of 3 μM bilirubin in PVSMCs. **(B)** Current-voltage (I-V) relationship of Cl_Ca_ currents in the absence and presence of bilirubin in PVSMCs (*n* = 7). **(C)** Expression level of TMEM16A mRNA in PVSMs after exposure to 30 μM bilirubin for 24 h (*n* = 9). Data are presented as means ± S.E.

As the plasma bilirubin level is increased in chronic liver diseases (Geerts et al., 2008), the effects of bilirubin on the expression and activity of TMEM16A channels were examined in murine PVSMCs. Under pCa 6.0 conditions in the pipette solution, whole-cell Cl_Ca_ currents were not affected by the application of 3 μM bilirubin to PVSMCs (26.2 ± 6.6 pA/pF at +100 mV, *n* = 7, *p* = 0.419 versus control, 19.4 ± 4.8 pA/pF, *n* = 7; Figures 6A,B). The expression of TMEM16A mRNA was unaltered by treatment with 30 μM bilirubin for 24 h in PVSMs (0.97 ± 0.02 of GAPDH, *n* = 9, *p* = 0.918 versus vehicle control, 1.00 ± 0.18, *n* = 9; Figure 6C). Therefore, bilirubin did not alter the expression or activity of TMEM16A channels in PVSMCs.

### Effects of Angiotensin II on Expression and Activity of TMEM16A Channels in Murine PVSMCs

**FIGURE 7:**
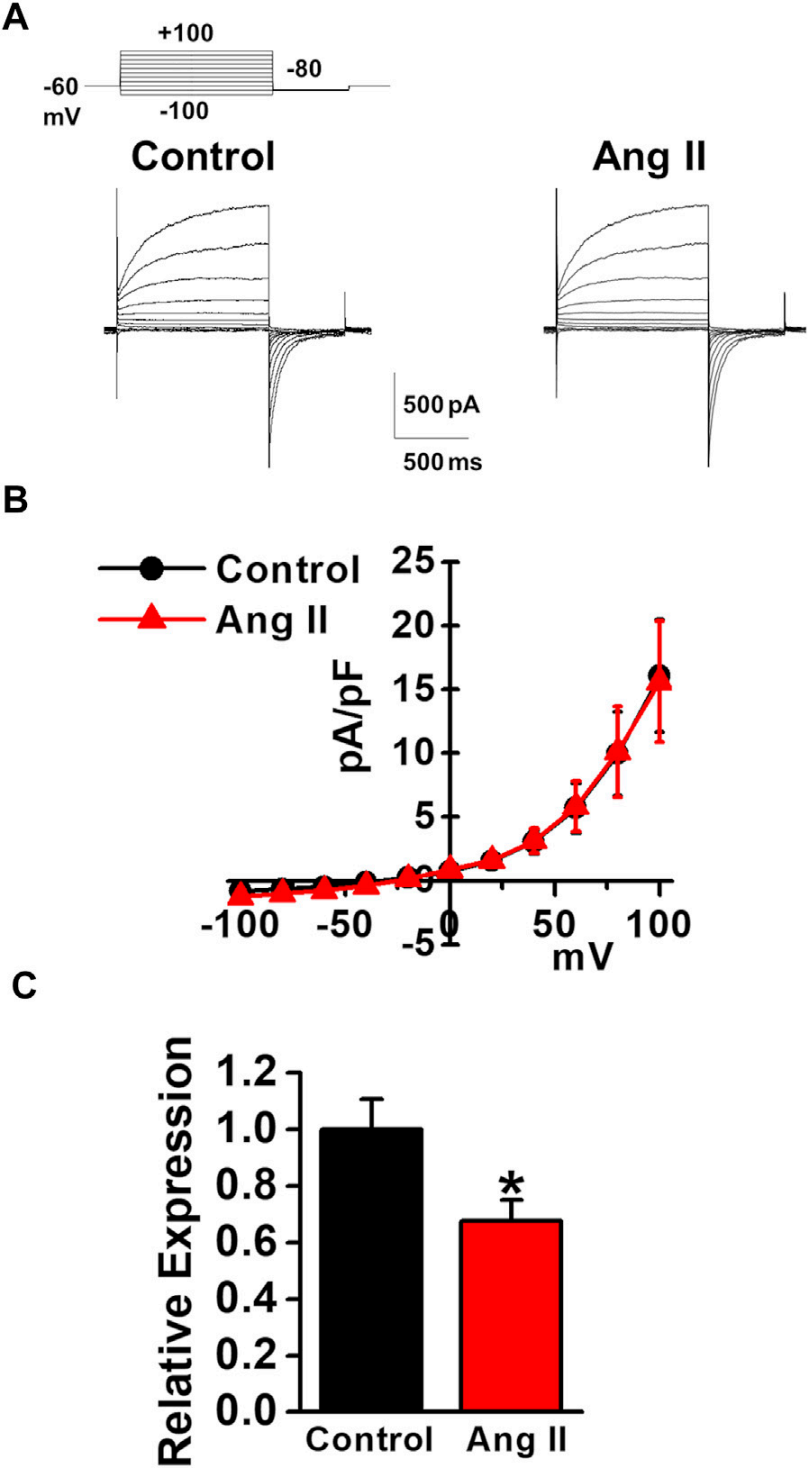
Effects of angiotensin II on TMEM16A expression and currents in murine PVSMCs. Effects of angiotensin II on the expression and activity of TMEM16A channels were examined in murine PVSMCs. **(A)** Representative traces of Cl_Ca_ currents in the absence and presence of 1 μM angiotensin II (Ang II) in PVSMCs. **(B)** Current-voltage (I-V) relationship of Cl_Ca_ currents in the absence and presence of angiotensin II in PVSMCs (*n* = 4). **(C)** Expression level of TMEM16A mRNA in PVSMs after the exposure to 1 μM angiotensin II for 24 h (*n* = 9–12). Data are presented as means ± S.E. *p < 0.05 versus vehicle control.

As the plasma angiotensin II level was reported to be increased in chronic liver diseases (Grace et al., 2012), the effects of angiotensin II on the expression and activity of TMEM16A channels were examined in murine PVSMCs. Under pCa 6.0 conditions in the pipette solution, whole-cell Cl_Ca_ currents were not affected by the application of 1 μM angiotensin II to PVSMCs (15.6 ± 4.7 pA/pF at +100 mV, *n* = 4, *p* = 0.946 versus control, 16.1 ± 4.4 pA/pF, *n* = 4; Figures 7A,B). Real-time PCR analysis revealed that the expression of TMEM16A mRNA was downregulated by the treatment with 1 μM angiotensin II for 24 h in PVSMs (0.68 ± 0.07 of GAPDH, *n* = 9, *p* = 0.032 versus vehicle control, 1.00 ± 0.11, *n* = 12; Figure 7C). This suggested that the expression of TMEM16A was downregulated by angiotensin II in PVSMCs.

## Discussion

Portal hypertension is a frequent major complication of cirrhosis. As the development of portal hypertension causes serious clinical symptoms such as gastroesophageal varices, variceal hemorrhage, splenomegaly, ascites, and hepatic encephalopathy, the underlying mechanism has been extensively investigated (Bosch et al., 2009; García-Pagán et al., 2012). In the present study, the pathological role of TMEM16A Cl_Ca_ channels in portal hypertension was focused on using cirrhotic BDL and non-cirrhotic PPVL mice. We found that 1) the expression of TMEM16A was downregulated in BDL-PVSMCs, but not in PPVL-PVSMCs, 2) the activity of Cl_Ca_ currents was reduced in BDL-PVSMCs, 3) the involvement of TMEM16A channels in spontaneous contractions was attenuated in BDL-PVSMs, and 4) the downregulation of TMEM16A expression in BDL-PVSMCs was partly induced by angiotensin II.

TMEM16A channels are ubiquitously expressed in epithelia, interstitial cells of Cajal (ICC), neurons, endothelium, and smooth muscles to regulate their functions (Pedemonte and Galietta, 2014). In vascular smooth muscles, TMEM16A Cl_Ca_ channels function in the regulation of membrane excitability, myogenic tone, and muscle contraction (Pedemonte and Galietta, 2014). In the present study, expression analyses of the TMEM16 family revealed that TMEM16A, TMEM16F, and TMEM16K genes were abundant in PVSMs, as reported previously (Ohshiro et al., 2014a). The most important finding was that the expression level of TMEM16A was lower in BDL-PVSMs (43%) than in sham-PVSMs, whereas the expression level in PPVL-PVSMCs was almost the same as that in sham-PVSMs. TMEM16F transcripts slightly decreased in BDL-PVSMs (18%). Although TMEM16F is necessary for the Ca^2+^-dependent phospholipid scramblase, it is hypothesized to form Cl^−^ channels. However, the activation of TMEM16F channels requires a higher concentration of [Ca^2+^]_cyt_ (EC_50_ of >10 μM) than TMEM16A channels (EC_50_ of 0.3 μM) (Pedemonte and Galietta, 2014). Therefore, TMEM16F channels may not function as Cl_Ca_ channels under the physiological conditions in PVSMCs. TMEM16K is not considered an ion channel (Pedemonte and Galietta, 2014). Taken together, the present study mainly focused on the pathophysiological functions of TMEM16A Cl_Ca_ channels in PVSMCs from cirrhotic portal hypertensive mice.

The amplitudes of peak and tail Cl_Ca_ currents were smaller in BDL-PVSMCs than in sham-PVSMCs. Our previous report showed that the Cl_Ca_ currents were inhibited by niflumic acid and T16A_inh_-A01 (Ohshiro et al., 2014a). The spontaneous contractions in sham-PVSMs were also blocked by T16A_inh_-A01. T16A_inh_-A01 may be somewhat less selectivity, but this suggests that TMEM16A Cl_Ca_ channel conductance is involved in the formation of spontaneous contractions, which play a significant role in the blood flow from mesenteric vascular beds to the liver. The spontaneous contractions in PVSMs depend on the activities of VDCCs and Cl_Ca_ channels, and are inhibited by niflumic acid (Kirkup et al., 1996; Saleh and Greenwood, 2005). In the present study, there was no significant difference in the expression level of VDCC subunits (α_1C_, β_2_, and β_3_) between sham- and BDL-PVSMs (data not shown). On the other hand, the sensitivity to T16A_inh_-A01 on spontaneous contractions in BDL-PVSMs was markedly lower (sham, 93% decrease versus BDL, 52% decrease in amplitude; and sham, 79% decrease versus BDL, 30% decrease in frequency). Taken together, these decreases in Cl_Ca_ channel components of currents and spontaneous contractions are consistent with the downregulation of TMEM16A expression in BDL-PVSMCs. In vascular smooth muscles, a decrease in Cl_Ca_ conductance shifts the resting membrane potential to the hyperpolarizing direction. This blocks Ca^2+^ influx through VDCCs, resulting in the attenuation of cell excitability in the form of Ca^2+^-dependent action potentials (Kitamura and Yamazaki, 2001; Bulley and Jaggar, 2014). Therefore, this may be a mechanism for protecting against increased portal pressure and related symptoms. However, the spontaneous contractions in sham-PVSMs were mostly inhibited by T16A_inh_-A01, whereas these in BDL-PVSMs were often observed even in the presence of T16A_inh_-A01, suggesting that the expression of other Cl_Ca_ channels was upregulated as a compensatory mechanism. Further experiments are necessary to elucidate the molecular switching of Cl_Ca_ channels in BDL-PVSMCs.

It has been reported that BDL-induced cirrhosis alters the expression level of ion channels such as voltage-dependent Na^+^ channels (Lee et al., 2016), epithelial Na^+^ channels (Kim et al., 2006), large-conductance K_Ca_ (BK_Ca_) channels (Yuan et al., 2016; Jadeja et al., 2017), ATP-sensitive K^+^ channels (Yuan et al., 2016), transient receptor potential canonical subfamily (TRPC) channels (Nedungadi and Cunningham, 2014; Jadeja et al., 2017), and transient receptor potential vanilloid subfamily (TRPV) channels (Nedungadi et al., 2012; Belghiti et al., 2013; Hong-Qian Wang et al., 2019). In addition, we noted the downregulation of TMEM16A (and also TMEM16F) expression in cirrhotic BDL mice, but not in non-cirrhotic PPVL mice. As both mouse models exhibited portal hypertrophy, the downregulation of TMEM16A expression in BDL-PVSMCs was not due to an increase in portal venous pressure. Rather, it may be caused by liver failure because the difference between BDL and PPVL mice is the presence and absence of fibrosis of the liver, respectively. In addition to PVSMCs, the downregulation of TMEM16A expression was observed in aortic smooth muscles from BDL mice (unpublished observation). Taken together, the downregulation may be mediated by endogenous compounds associated with cirrhosis, but not by portal hypertension.

Some endogenous substances are released into the blood from damaged liver cells. We focused on bilirubin (Geerts et al., 2008) and angiotensin II (Grace et al., 2012), which are known to be increased in the blood in cirrhosis. Bilirubin regulates the expression and activity of voltage-dependent Na^+^ channels (Shi et al., 2019), epithelial Na^+^ channels (Xue-Ping Wang et al., 2019), acid-sensing ion channels (ASICs) (Lai et al., 2020), and VDCCs (Liang et al., 2017; Albanna et al., 2019). Although we examined the effects of bilirubin on the expression and activity of TMEM16A channels, it had no effects in PVSMCs. On the other hand, angiotensin II down-/up-regulates vascular ion channels such as VDCCs (Kharade et al., 2013), voltage-dependent K^+^ channels (Barrese et al., 2018), BK_Ca_ channels (Nieves-Cintrón et al., 2007), and transient receptor potential melastatin subfamily (TRPM) channels (He et al., 2005; Huang et al., 2017). In addition, angiotensin II downregulates TMEM16A functions in rat basilar smooth muscle cells from 2K2C renohypertensive rats (Wang et al., 2012) and TMEM16A expression in human aortic smooth muscle cells (Zhang et al., 2015), whereas it upregulates TMEM16A functions in aorta and mesenteric arterial smooth muscle cells from spontaneous hypertensive rats (SHR) (Wang et al., 2015). Our study demonstrated that angiotensin II, the plasma concentration of which increases in patients with cirrhotic portal hypertension from 20 to 200 pg/ml (Grace et al., 2012), downregulates the TMEM16A expression in murine PVSMCs. As angiotensin II did not affect Cl_Ca_ currents, it did not interact directly with TMEM16A channels in PVSMCs. Thus, the underlying mechanism is likely to through indirect action such as genomic effects. The expression level of TMEM16A channels is altered in cardiovascular diseases such as systemic hypertension (Wang et al., 2012; Wang et al., 2015), pulmonary hypertension (Forrest et al., 2012; Sun et al., 2012; Papp et al., 2019), and diabetic gastroparesis (Mazzone et al., 2011). In addition, cirrhosis induces the downregulation of TMEM16A expression in vascular smooth muscle cells.

We demonstrated that TMEM16A Cl_Ca_ channels play a role in vascular tone and contraction in PVSMCs from the cirrhotic portal hypertensive model. More recently, similar downregulation was observed in the same preparation, but it was involved in cell proliferation (Zeng et al., 2018). The activity of TMEM16A channels was suggested to be involved in cell survival and death, in addition to smooth muscle contraction (Pedemonte and Galietta, 2014). Although TMEM16A channels can activate Ca^2+^/calmodulin-dependent protein kinase II (CaMKII) and mitogen-activated protein kinase (MAPK) pathways (Wang et al., 2017), the signaling mechanism of TMEM16A channels for the proliferation and migration of smooth muscle cells remains unclear. Taken together, TMEM16A Cl_Ca_ channels play an important role in both the regulation of vascular tone and remodeling in cirrhotic portal hypertension. On the other hand, the pathological mechanism of non-cirrhotic portal hypertension remains unclear in the present study. Further studies using PPVL mice are necessary because there are no specific drugs for the treatment of idiopathic portal hypertension currently.

In conclusion, the expression and activity of TMEM16A channels were downregulated in PVSMCs from BDL mice associated with cirrhosis. This downregulation is partly mediated by increased angiotensin II in cirrhosis. Currently, non-selective β blockers, vasopressin analogues, and somatostatin analogues are commonly used to prevent portal hypertensive bleeding in cirrhosis. Novel therapeutic targets have been discussed as treatment options for portal hypertension; however, these strategies are far from satisfactory (Schwabl and Laleman, 2017). Our study revealed the involvement of TMEM16A Cl_Ca_ channels in the pathological mechanism underlying cirrhotic portal hypertension and provides a novel therapeutic target.

## Data Availability

The datasets presented in this study can be found in online repositories. The names of the repository/repositories and accession number(s) can be found in the article/Supplementary Material.
